# Chain Hexagonal Cacti with the Extremal Eccentric Distance Sum

**DOI:** 10.1155/2014/897918

**Published:** 2014-03-10

**Authors:** Hui Qu, Guihai Yu

**Affiliations:** School of Mathematics, Shandong Institute of Business and Technology, 191 Binhaizhong Road, Yantai, Shandong 264005, China

## Abstract

Eccentric distance sum (EDS), which can predict biological and physical properties, is a topological index based on the eccentricity of a graph. In this paper we characterize the chain hexagonal cactus with the minimal and the maximal eccentric distance sum among all chain hexagonal cacti of length *n*, respectively. Moreover, we present exact formulas for EDS of two types of hexagonal cacti.

## 1. Introduction

Topological index, which can be used to characterize some property of the molecule graph, is a numeric and invariant quantity of a structure graph under graph isomorphism. Recently a novel graph invariant for predicting biological and physical properties—*eccentric distance sum*—was introduced by Gupta eta al. [1]. It has a vast potential in structure activity/property relationships. The authors [1] have shown that some structure activity and quantitative structure-property studies using eccentric distance sum were better than the corresponding values obtained using the Wiener index.

Throughout this paper we only consider simple connected graphs. Let *G* be a simple connected graph with the vertex set *V*(*G*). For vertices *u*, *v* ∈ *V*(*G*), the* distance d*(*u*, *v*) is defined as the length of the shortest path between *u* and *v* in *G*; *D*_*G*_(*v*) (or *D*(*v*) for short) denotes the sum of distances from *v*. The* eccentricity ε*_*G*_(*v*) (or *ε*(*v*) for short) of a vertex *v* is the maximum distance from *v* to any other vertex in *G*. The eccentric distance sum (EDS) of *G* is defined as

(1) $$\begin{array}{c} \xi^{d}\left(G\right)=\sum_{v\in V(G)}\varepsilon_{G}\left(v\right)D_{G}\left(v\right). \end{array}$$

In [2] authors investigated the eccentric distance sum of unicyclic graphs with given girth and characterize the extremal graphs with the minimal and the second minimal EDS; they also characterize the trees with the minimal EDS among the *n*-vertex trees of a given diameter. Iliç et al. [3] studied the various lower and upper bounds for the EDS in terms of other graph invariants including the Wiener index, the degree distance, the eccentric connectivity index, independence number, connectivity, matching number, chromatic number, and clique number. Zhang and Li [4] considered the maximal eccentric distance sum of graphs and determined the *n*-vertex trees with the first four maximal EDS. Also they characterized the *n*-vertex unicyclic graphs with the first three maximal EDS. Li et al. [5] investigated the trees with the minimal and second minimal EDS among *n*-vertex trees with given matching number; as a continuance they also determine the trees with the third and fourth minimal EDS among the *n*-vertex trees and characterized the trees with the second EDS among the *n*-vertex trees of a given diameter. For other recent results on EDS, the readers are referred to [6, 7].

A* cactus graph* is a connected graph in which no edges lie in more than one cycle. Consequently, each block of a cactus graph is either an edge or a cycle. If all blocks of a cactus *G* are cycles of the same length *m*, the cactus is *m*-uniform. A hexagonal cactus is a 6-uniform cactus, that is, a cactus in which every block is a hexagon. A vertex shared by two or more hexagons is called a* cut vertex*. If each hexagon of a hexagonal cactus *G* has at most two cut vertices and each cut vertex is shared by exactly two hexagons, we call *G* as a* chain hexagonal cactus*. The number of hexagons in *G* is called the* length* of the chain. Evidently, there exit exactly two hexagons which share only one cut vertex in any chain hexagonal cactus of length greater than one. Such hexagons are called* terminal hexagons*, and other remaining hexagons are called* internal hexagons*. Let *ℭ*_*n*_ be the set of all chain hexagonal cacti of length *n*.

Let *u* and *v* be two vertices in *C*_6_. They are said to be in* orthoposition* if they are adjacent in *C*_6_. If the distance between *u* and *v* is two, they are in* metaposition*. They are in* paraposition* if the distance between them is three. An internal hexagon in a chain hexagonal cactus is called* orthohexagon*,* matahexagon*, or* parahexagon* if its cut vertices are in ortho-, meta-, or paraposition, respectively. A chain hexagonal cactus of length *n* is called an* orthochain* if all its internal hexagons are orthohexagons, denoted by *O*_*n*_. The* metachain* and* parachain* of length *n* are defined in a completely analogous manner, denoted by *M*_*n*_, *L*_*n*_, respectively.

Recently, Došlić and Måløy [8] considered the chain hexagonal cacti and derived explicit recurrences for their matching and independence polynomials and explicit recurrences for the number of matchings and independence set of certain types. The present paper was motivated by [8]; we investigate the eccentric distance sum of chain hexagonal cactus of length *n* and characterize the chain hexagonal cacti with the minimal and the maximal eccentric distance sums among all chain hexagonal cacti of length *n*(>2), respectively.

## 2. The Minimal and the Maximal EDS of Chain Hexagonal Cacti

In this section we determine the chain hexagonal cacti with the maximum and minimum EDS. In addition, we give exact formulas for EDS of two types of hexagonal cacti.

**Lemma 1**

**Figure 1:**
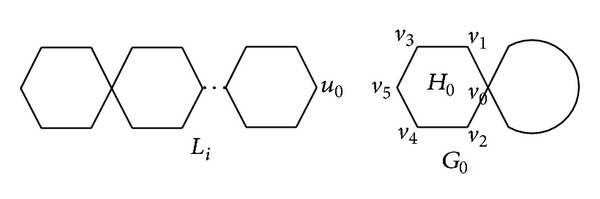
Two chain hexagonal cacti *L*_*i*_ and *G*_0_.

Let *L*_*i*_ be a parachain of lengths *n*_0_ (*n*_0_ ≥ 1) and *u*_0_ ∈ *V*(*L*_*i*_) (see Figure 1). Let *G*_0_ be a chain hexagonal cactus of length *n* − *n*_0_ and *v*_*j*_ ∈ *V*(*G*_0_)  (*j* = 0,1, 2,3, 4,5) (see Figure 1). Let *G*_1_ be the chain hexagonal cactus of length *n* obtained from *L*_*i*_ and *G*_0_ by identifying *u*_0_ with *v*_1_. Let *G*_2_ be the graph obtained from *L*_*i*_ and *G*_0_ by identifying *u*_0_ with *v*_3_. Let *G*_3_ be the graph obtained from *L*_*i*_ and *G*_0_ by identifying *u*_0_ with *v*_5_. If *n*_0_ ≤ ⌊(*n* − 1)/2⌋, then

(2) $$\begin{array}{c} \xi^{d}\left(G_{1}\right)<\xi^{d}\left(G_{2}\right)<\xi^{d}\left(G_{3}\right). \end{array}$$

**Proof**

Let *H*_0_ be the hexagon consisting of vertices *v*_*i*_ (*i* = 0,1,…, 5) in *G*_0_ (see Figure 1). By the definition of eccentric distance sum, we have

(3) ξd(Gi)=∑v∈V(Li−u0)εGi(v)DGi(v)+∑v∈V(H0)εGi(v)DGi(v)+∑v∈V(G0−H0)εGi(v)DGi(v), (i=1,2,3).

Let *A*_*i*_, *B*_*i*_, and *C*_*i*_ (*i* = 1,2, 3) denote the three terms of right equality above, respectively. We only need to prove the following three inequalities:  *A*_3_ > *A*_2_ > *A*_1_, *B*_2_ + *C*_2_ > *B*_1_ + *C*_1_, and *B*_3_ + *C*_3_ > *B*_2_ + *C*_2_.

For any *v* ∈ *V*(*L*_*i*_ − *u*_0_), we have

(4) εG1(v)<εG2(v)<εG3(v),∑u∈V(Li−u0)dG1(u,v)=∑u∈V(Li−u0)dG2(u,v)=∑u∈V(Li−u0)dG3(u,v),∑u∈V(H0)dG1(u,v)=∑u∈V(H0)dG2(u,v)=∑u∈V(H0)dG3(u,v),∑u∈V(G0−H0)dG1(u,v)<∑u∈V(G0−H0)dG2(u,v)<∑u∈V(G0−H0)dG3(u,v),

which yield that *A*_1_ < *A*_2_ < *A*_3_.

For any *v* ∈ *V*(*G*_0_), it is evident that *ε*_*G*_1__(*v*) ≤ *ε*_*G*_2__(*v*) ≤ *ε*_*G*_3__(*v*). Moreover,

(5) $$\begin{array}{c} D_{G_{i}}\left(v\right)=\sum_{u\in V(G_{0})}d_{G_{i}}\left(u,v\right)+\sum_{u\in V(L_{i}-u_{0})}d_{G_{i}}\left(u,v\right). \end{array}$$

Note that ∑_*u*∈*V*(*G*_0_)_ *d*_*G*_1__(*u*, *v*) = ∑_*u*∈*V*(*G*_0_)_ *d*_*G*_2__(*u*, *v*) = ∑_*u*∈*V*(*G*_0_)_ *d*_*G*_3__(*u*, *v*). For the part ∑_*u*∈*V*(*L*_*i*_−*u*_0_)_ *d*_*G*_*i*__(*u*, *v*), we divide into two cases to deal with it.

*Case 1 *(*v* ∈ *V*(*G*_0_ − *H*_0_)). Consider the following:

(6) ∑u∈V(Li−u0)dG2(u,v)=∑u∈V(Li−u0)(dG1(u,v)+1)=5n+∑u∈V(Li−u0)dG1(u,v),∑u∈V(Li−u0)dG3(u,v)=∑u∈V(Li−u0)(dG1(u,v)+2)=10n+∑u∈V(Li−u0)dG1(u,v).

*Case 2* (*v* ∈ *V*(*H*_0_) = {*v*_0_, *v*_1_, *v*_2_, *v*_3_, *v*_4_, *v*_5_}). Consider the following:

(7) ∑u∈V(Li−u0)dG2(u,v0)=∑u∈V(Li−u0)(dG1(u,v0)+1)=5n+∑u∈V(Li−u0)dG1(u,v0),∑u∈V(Li−u0)dG3(u,v0)=∑u∈V(Li−u0)(dG1(u,v0)+2)=10n+∑u∈V(Li−u0)dG1(u,v0),∑u∈V(Li−u0)dG2(u,v1)=∑u∈V(Li−u0)(dG1(u,v1)+1)=5n+∑u∈V(Li−u0)dG1(u,v1),∑u∈V(Li−u0)dG3(u,v1)=∑u∈V(Li−u0)(dG1(u,v1)+2)=10n+∑u∈V(Li−u0)dG1(u,v1),∑u∈V(Li−u0)dG2(u,v2)=∑u∈V(Li−u0)(dG1(u,v2)+1)=5n+∑u∈V(Li−u0)dG1(u,v2),∑u∈V(Li−u0)dG3(u,v2)=∑u∈V(Li−u0)dG1(u,v2),∑u∈V(Li−u0)dG2(u,v3)=∑u∈V(Li−u0)(dG1(u,v3)−1)=−5n+∑u∈V(Li−u0)dG1(u,v3),∑u∈V(Li−u0)dG3(u,v3)=∑u∈V(Li−u0)dG1(u,v3),∑u∈V(Li−u0)dG2(u,v4)=∑u∈V(Li−u0)(dG1(u,v4)−1)=−5n+∑u∈V(Li−u0)dG1(u,v4),∑u∈V(Li−u0)dG3(u,v4)=∑u∈V(Li−u0)(dG1(u,v4)−2)=−10n+∑u∈V(Li−u0)dG1(u,v4),∑u∈V(Li−u0)dG2(u,v5)=∑u∈V(Li−u0)(dG1(u,v5)−1)=−5n+∑u∈V(Li−u0)dG1(u,v5),∑u∈V(Li−u0)dG3(u,v5)=∑u∈V(Li−u0)(dG1(u,v5)−2)=−10n+∑u∈V(Li−u0)dG1(u,v5).

Therefore, we have

(8) C2−C1 =∑v∈V(G0−H0)εG2(v)DG2(v)−∑v∈V(G0−H0)εG1(v)DG1(v) ≥∑v∈V(G0−H0)εG2(v)[DG2(v)−DG1(v)] =∑v∈V(G0−H0)εG2(v)[∑u∈V(Li−u0)dG2(u,v)−∑u∈V(Li−u0)dG1(u,v)] ≥∑v∈V(G0−H0)5n·εG1(v) ≥25n(n−n0−1)·min⁡⁡{εG1(v) ∣ v∈V(G0−H0)} ≥25n(n−n0−1)(n−n0+2).

Similarly, we have *C*_3_ − *C*_2_ ≥ 25*n*(*n* − *n*_0_ − 1)(*n* − *n*_0_ + 2).

For *v*_*i*_ ∈ *V*(*H*_0_)  (*i* = 0,1, 2,3, 4,5), it follows that

(9) DG2(v0)=∑u∈V(G0)dG2(u,v0)+∑V(Li−u0)dG2(u,v0)=∑u∈V(G0)dG1(u,v0)+∑V(Li−u0)dG1(u,v0)+5n=DG1(v0)+5n, DG2(v1)=DG1(v1)+5n, DG2(v2)=DG1(v2)+5n, DG2(v3)=DG1(v3)−5n, DG2(v4)=DG1(v4)−5n, DG2(v5)=DG1(v5)−5n.

So we have

(10) B2=∑v∈V(H0)εG2(v)DG2(v)=εG2(v0)DG2(v0)+εG2(v1)DG2(v1) +εG2(v2)DG2(v2)+εG2(v3)DG2(v3) +εG2(v4)DG2(v4)+εG2(v5)DG2(v5)≥εG1(v0)(DG1(v0)+5n)+εG1(v1)(DG1(v1)+5n) +εG1(v2)(DG1(v2)+5n)+εG1(v3)(DG1(v3)−5n) +εG1(v4)(DG1(v4)−5n)+εG1(v5)(DG1(v5)−5n)=B1+5nεG1(v0)+5nεG1(v1)+5nεG1(v2) −5nεG1(v3)−5nεG1(v4)−5nεG1(v5)=B1+5n[εG1(v0)+(εG1(v0)+1)+(εG1(v0)+1)−(εG1(v0)+2)−(εG1(v0)+2)−(εG1(v0)+3)]=B1−25n.

Similarly, we can get

(11) B3−B2≥εG1(v0)·(5n)+εG1(v1)·(5n) +εG1(v2)·(−5n)+εG1(v3)·(5n) +εG1(v4)·(−5n)+εG1(v5)·(−5n)=5n(εG1(v0)+εG1(v0)+1−εG1(v0)−1   +εG1(v0)+2−εG1(v0)−2−εG1(v0)−3)=−15n.

So we have

(12) $$\begin{array}{c} B_{2}+C_{2}-B_{1}-C_{1}\geq 25n\left(n-n_{0}-1\right)\left(n-n_{0}+2\right)-25n>0,\\ B_{3}+C_{3}-B_{2}-C_{2}\geq 25n\left(n-n_{0}-1\right)\left(n-n_{0}+2\right)-15n>0. \end{array}$$

We complete the proof.

By Lemma 1, we can get the following result.

**Theorem 2**

Let *G*_*n*_ be a chain hexagonal cactus of length *n*. Then

(13) $$\begin{array}{c} \xi^{d}\left(O_{n}\right)\leq \xi^{d}\left(G_{n}\right)\leq \xi^{d}\left(L_{n}\right). \end{array}$$

In the following, we will calculate the values of *ξ* ^*d*^(*O*_*n*_) and *ξ* ^*d*^(*L*_*n*_).

**Theorem 3**

Consider

(14) $$\begin{array}{c} \xi^{d}\left(L_{n}\right)=\{\begin{array}{cc} \frac{1}{32}n(1875n^{3}+1640n^{2} & \\ \;\;\;+956n-80) & if\;\;n\;\;is\;\;even,\\ \frac{1}{32}(1875n^{4}+1260n^{3} & \\ \;\;+974n^{2}-12n-65) & if\;\;n\;\;is\;\;odd, \end{array}\\ \xi^{d}\left(O_{n}\right)=\{\begin{array}{cc} \frac{1}{96}n(625n^{3}+7300n^{2} & \\ \;\;\;+19988n-8032) & if\;\;n\;\;is\;\;even,\\ \frac{1}{96}(625n^{4}+7300n^{3} & \\ \;\;+20138n^{2}-6532n-795) & if\;\;n\;\;is\;\;odd. \end{array} \end{array}$$

For some *v* ∈ *V*(*H*_*k*_), assume that *d*(*v*, *v*_*k*_) = *y* and *d*(*v*, *v*_*k*+1_) = *z*. Then we have

(15) D(v)=∑u∈V(H1)d(u,v)+⋯+∑u∈V(Hk)d(u,v) +⋯+∑u∈V(Hn)d(u,v)−∑j=2nd(vj,v)=∑u∈V(H1)(d(u,v2)+d(v2,v)) +⋯+∑u∈V(Hj);j<k(d(u,vj+1)+d(vj+1,v)) +⋯+∑u∈V(Hk)d(u,v) +⋯+∑u∈V(Hj);j>k(d(u,vj)+d(vj,v)) +⋯+∑u∈V(Hn)(d(u,vn)+d(vn,v))−∑j=2nd(vj,v)=(9+∑u∈V(H1)d(v2,v)) +⋯+(9+∑u∈V(Hj);j<kd(vj+1,v)) +⋯+9+⋯+(9+∑u∈V(Hj);j>kd(vj,v)) +⋯+(9+∑u∈V(Hn)d(vn,v))−∑j=2nd(vj,v)=9n+∑u∈V(H1)[y+3(k−2)] +⋯+∑u∈V(Hj);j<k[y+3(k−(j+1))] +⋯+∑u∈V(Hk−1)[y+3(k−k)]+∑u∈V(Hk+1)[z+0] +∑u∈V(Hk+2)[z+3(k+2−(k+1))] +⋯+∑u∈V(Hj);j>k[z+3(j−(k+1))] +⋯+∑u∈V(Hn)[z+3(n−(k+1))]−∑j=2nd(vj,v)=9n+5∑j=1k−1[y+3(k−(j+1))] +5∑j=k+1n[z+3(j−(k+1))].

**Figure 2:**
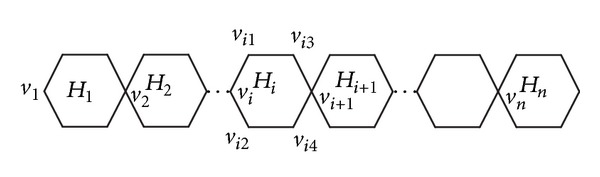
The parachain *L*_*n*_.

For some hexagon *H*_*i*_ (*i* ≤ *n*) (see Figure 2) in *L*_*n*_, the eccentricity of every vertex on *H*_*i*_ can be derived as follows:

(16) $$\begin{array}{c} \varepsilon \left(v_{i}\right)=3\left(n+1-i\right),\\ \varepsilon \left(v_{i1}\right)=3\left(n+1-i\right)-1,\\ \varepsilon \left(v_{i2}\right)=3\left(n+1-i\right)-1,\\ \varepsilon \left(v_{i3}\right)=3\left(n+1-i\right)-2,\\ \varepsilon \left(v_{i4}\right)=3\left(n+1-i\right)-2,\\ \varepsilon \left(v_{i+1}\right)=3\left(n-i\right). \end{array}$$

When *n* is even, we have

(17) ξd(Ln)=∑i=1n(∑v∈Hiε(v)D(v))−∑i=2nε(vi)D(vi)=2∑i=1n/2(∑v∈Hiε(v)D(v))−∑i=2nε(vi)D(vi)=2∑i=1n/2{3(n+1−i)[9n+5∑j=1i−1[0+3(i−(j+1))]+5∑j=i+1n[3+3(j−(i+1))]]+2[[3(n+1−i)−1]×[9n+5∑j=1i−1[1+3(i−(j+1))]+5∑j=i+1n[2+3(j−(i+1))]]]+2[[3(n+1−i)−2]×[9n+5∑j=1i−1[2+3(i−(j+1))]+5∑j=i+1n[1+3(j−(i+1))]]]+3(n−i)[9n+5∑j=1i−1‍[3+3(i−(j+1))]+5∑j=i+1n[0+3(j−(i+1))]]} −2∑i=2n/23(n+1−i)[9n+5∑j=1i−1[0+3(i−(j+1))]+5∑j=i+1n[3+3(j−(i+1))]] −3·n2[9n+5∑j=1n/2[0+3(n2+1−(j+1))]+5∑j=(n/2)+2n[3+3(j−(n2+2))]]=132n(1875n3+1640n2+956n−80).

When *n* is odd, we have

(18) ξd(Ln)=∑i=1n(∑v∈Hiε(v)D(v))−∑i=2nε(vi)D(vi)=2∑i=1(n−1)/2(∑v∈Hiε(v)D(v))+∑v∈H(n+1)/2ε(v)D(v) −∑i=2nε(vi)D(vi)=2∑i=1(n−1)/2{3(n+1−i)[9n+5∑j=1i−1[0+3(i−(j+1))]+5∑j=i+1n[3+3(j−(i+1))]]+2[3(n+1−i)−1]×[9n+5∑j=1i−1[1+3(i−(j+1))]+5∑j=i+1n[2+3(j−(i+1))]]+2[3(n+1−i)−2]×[9n+5∑j=1i−1[2+3(i−(j+1))]+5∑j=i+1n[1+3(j−(i+1))]]+3(n−i)×[9n+5∑j=1i−1[3+3(i−(j+1))]+5∑j=i+1n[0+3(j−(i+1))]]} +2·3·n+12[9n+5∑j=1(n−1)/2[0+3(n+12−(j+1))]+5∑j=(n+3)/2n[3+3(j−(n+12+1))]] +2·2·[3(n+12)−1] ×[9n+5∑j=1(n−1)/2[1+3(n+12−(j+1))]   +5∑j=(n+3)/2n[2+3(j−(n+12+1))]] −2∑i=2(n+1)/23(n+1−i)[9n+5∑j=1i−1[0+3(i−(j+1))]+5∑j=i+1n[3+3(j−(i+1))]]=132(1875n4+1260n3+974n2−12n−65).

Let *H*_*k*_ be a hexagon in *O*_*n*_. For some *v* ∈ *V*(*H*_*k*_), assume that *d*(*v*, *v*_*k*_) = *y* and *d*(*v*, *v*_*k*+1_) = *z*. Similar to the case in *L*_*n*_, we have

(19) D(v)=∑i=1n ‍∑u∈V(Hi)d(u,v)−∑j=2nd(vj,v)=9n+5∑j=1i−1[y+i−(j+1)] +5∑j=i+1n[z+j−(i+1)].

**Figure 3:**
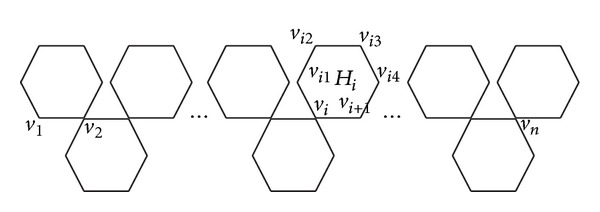
The orthochain *O*_*n*_.

For some hexagon *H*_*i*_  (*i* < (*n* + 1)/2) (see Figure 3), the eccentricities of every vertex on *H*_*i*_ can be derived as follows:

(20) $$\begin{array}{c} \varepsilon \left(v_{i1}\right)=4+n-i,\\ \varepsilon \left(v_{i2}\right)=5+n-i,\\ \varepsilon \left(v_{i3}\right)=4+n-i,\\ \varepsilon \left(v_{i4}\right)=3+n-i,\\ \varepsilon \left(v_{i}\right)=3+n-i,\\ \varepsilon \left(v_{i+1}\right)=2+n-i. \end{array}$$

When *n* is even, then we have

(21) ξd(On)=∑i=1n(∑v∈Hiε(v)D(v))−∑i=2nε(vi)D(vi)=2∑i=1n/2(∑v∈Hiε(v)D(v))−∑i=2nε(vi)D(vi)=2∑i=1n/2{(4+n−i)[9n+5∑j=1i−1[1+i−(j+1)]‍+5∑j=i+1n[2+j−(i+1)]]+(5+n−i)[9n+5∑j=1i−1[2+i−(j+1)]+5∑j=i+1n[3+j−(i+1)]]+(4+n−i)[9n+5∑j=1i−1[3+i−(j+1)]+5∑j=i+1n[2+j−(i+1)]]+(3+n−i)[9n+5∑j=1i−1[2+i−(j+1)]+5∑j=i+1n[1+j−(i+1)]]+(3+n−i)[9n+5∑j=1i−1[0+i−(j+1)]+5∑j=i+1n[1+j−(i+1)]]+(2+n−i)[9n+5∑j=1i−1[1+i−(j+1)]+5∑j=i+1n[0+j−(i+1)]]} −2∑i=2n/2(n+3−i)[9n+5∑j=1i−1[0+i−(j+1)]+5∑j=i+1n[1+j+(i+1)]] −[n+3−(n2+1)] ×[9n+5∑j=1n/2[0+n2+1−(j+1)]+5∑j=(n/2)+2n[1+j−(n2+2)]]=196n(625n3+7300n2+19988n−8032).

When *n* is odd, then we have

(22) ξd(On)=∑i=1n(∑v∈Hiε(v)D(v))−∑i=2nε(vi)D(vi)=2∑i=1(n−1)/2(∑v∈Hiε(v)D(v))+∑v∈H(n+1)/2ε(v)D(v) −∑i=2nε(vi)D(vi)=2∑i=1(n−1)/2{(4+n−i)[9n+5∑j=1i−1[1+i−(j+1)]‍+5∑j=i+1n[2+j−(i+1)]]+(5+n−i)[9n+5∑j=1i−1[2+i−(j+1)]+5∑j=i+1n[3+j−(i+1)]]+(4+n−i)[9n+5∑j=1i−1[3+i−(j+1)]+5∑j=i+1n[2+j−(i+1)]]+(3+n−i)[9n+5∑j=1i−1[2+i−(j+1)]+5∑j=i+1n[1+j−(i+1)]]+(3+n−i)[9n+5∑j=1i−1[0+i−(j+1)]+5∑j=i+1n[1+j−(i+1)]]+(2+n−i)[9n+5∑j=1i−1[1+i−(j+1)]+5∑j=i+1n[0+j−(i+1)]]} +2·(4+n−n+12) ×[9n+5∑j=1(n−1)/2[1+n+12−(j+1)]   +5∑j=(n+3)/2n[2+j−(n+32)]] +2·(5+n−n+12) ×[9n+5∑j=1(n−1)/2[2+n+12−(j+1)]   +5∑j=(n+3)/2n[3+j−(n+32)]] +2·(3+n−n+12) ×[9n+5∑j=1(n−1)/2[0+n+12−(j+1)]   +5∑j=(n+3)/2n[1+j−(n+32)]] −2∑i=2(n+1)/2(n+3−i) ×[9n+5∑j=1i−1[0+i−(j+1)]   +5∑j=i+1n[1+j−(i+1)]]=196(625n4+7300n3+20138n2−6532n−795).
